# The Effects of Microgravity on Differentiation and Regeneration in Neural Stem Cells

**DOI:** 10.3390/cells15040371

**Published:** 2026-02-20

**Authors:** Qiuyan Hao, Hao Tian, Na Lv, Fengtang Yang, Hui Zhen, Zhonghong Cao

**Affiliations:** School of Life Science and Medicine, Shandong University of Technology, No.266 Xincun West Road, Zhangdian District, Zibo 255000, China; 19861607857@163.com (Q.H.); htian@sdut.edu.cn (H.T.); 17685639096@163.com (N.L.); fengtangyang@163.com (F.Y.)

**Keywords:** neural stem cells (NSCs), development, regeneration, gene expression, signal pathway, microgravity

## Abstract

**Highlights:**

**What are the main findings?**

- In scientific research, perspectives diverge on microgravity’s impact on neural stem cell (NSC) development, with some studies indicating promotion and others inhibition.
- This review clarifies the key features of NSCs, examines the contrasting effects of microgravity on their development and regeneration (both promoting and inhibiting), and explores the underlying molecular and cellular mechanisms. These insights offer valuable guidance for NSC-based therapies in space and regenerative medicine.

**What are the implications of the main findings?**

- These findings are expected to inspire novel approaches for studying neural systems pertinent to space medicine while facilitating the optimization of NSC applications in regenerative medicine.

**Abstract:**

Neural stem cells (NSCs) are self-renewing, multipotent cells of the central nervous system (CNS) that can differentiate into a range of specialized cell types, including neurons, astrocytes, and oligodendrocytes (OLs). Due to their remarkable ability to self-renew and differentiate, NSCs hold immense potential for the treatment of neurodegenerative diseases (NDDs). However, clinical translation remains hindered by challenges such as expansion difficulties and phenotypic drift. This review synthesizes evidence on the divergent effects of microgravity on NSC biology. While real spaceflight has been shown to enhance NSC proliferation, it paradoxically reduces neurosphere volume. Microgravity simulations yield contrasting results: rotating wall vessel (RWV) systems promote neuron and astrocyte generation, whereas rotating cell culture systems (RCCSs) inhibit differentiation despite the use of pro-differentiation media. These phenotypic variations critically depend on experimental conditions, cell sources, and observation time. Future research should focus on elucidating cross-pathway interactions and optimizing culture parameters to enable clinical-scale NSC applications.

## 1. Introduction

Neural stem cells (NSCs) are adult stem cells with the remarkable ability to self-renew and exhibit multipotent differentiation. During embryonic development, NSCs are broadly dispersed throughout the brain. In contrast, in adult mammals, their distribution becomes restricted to specific regions: the sub-ventricular zone (SVZ) and the sub-granular zone (SGZ) of the dentate gyrus [1,2]. As central nervous system (CNS) cells, NSCs can self-renew and differentiate into neurons, astrocytes, and oligodendrocytes (OLs) [3]. These unique properties position NSCs as promising therapeutic candidates for CNS diseases [4,5,6].

Neurological disorders encompass a wide range of conditions, including neurodegenerative diseases (NDDs) such as Parkinson’s and Alzheimer’s, as well as spinal cord injuries. Among these, NDDs are particularly prevalent and debilitating. It is projected that within the next 20 years, NDDs will become the second leading cause of human mortality, surpassed only by cardiovascular disease [7]. Despite their immense therapeutic potential, NSCs face significant barriers to clinical translation. These challenges include difficulties in expanding NSCs in culture and phenotypic drift during prolonged cultivation [8,9,10]. Traditional two-dimensional (2D) culture systems fail to recapitulate the complex three-dimensional (3D) tissue microenvironment, resulting in compromised cell quality and therapeutic efficacy [11].

Critically, these limitations directly hinder the clinical application of NSCs for treating NDDs and other neurological conditions. Recent advances in space medicine have revealed that microgravity research may offer unprecedented opportunities to overcome these translational barriers and accelerate therapeutic development [12,13,14].

Microgravity provides a distinctive alternative that may address these challenges. Achievable through orbital missions or ground-based simulations [15,16], microgravity offers a unique environment for studying cellular adaptation beyond traditional culture conditions. Exposure to microgravity induces profound morphological and molecular adaptations in human cells [17,18,19,20,21,22,23,24]. However, opinions on how microgravity affects brain stem cells continue to differ. On one hand, microgravity-exposed NSCs exhibit increased cell diameter, enhanced proliferation, and accelerated cell cycling [25]. On the other hand, others demonstrate inhibited proliferation and reduced neurosphere volume under microgravity conditions [26]. To elucidate these divergent effects, this review synthesizes current evidence on microgravity’s impact on NSC development, regeneration capacity, and underlying regulatory mechanisms. It also aims to provide insights for NSC clinical translation in regenerative medicine and interdisciplinary perspectives on neural mechanisms in space medicine.

## 2. Neural Stem Cells

In 1992, scientists first isolated a cell population from the striatum of mice that could continuously divide and proliferate in vitro and differentiate along multiple lineages [27], establishing the groundwork for the study of NSCs. The concept of NSCs was further refined in 1997 when McKay characterized them not merely as cells present in the nervous system, but as cells with the capacity for self-renewal and the potential to generate neurons, glial cells, and oligodendrocytes throughout the CNS [28]. These cells not only play an important role in the normal development of the brain and spinal cord, but they are also essential for nervous system regeneration and repair.

**Figure 1:**
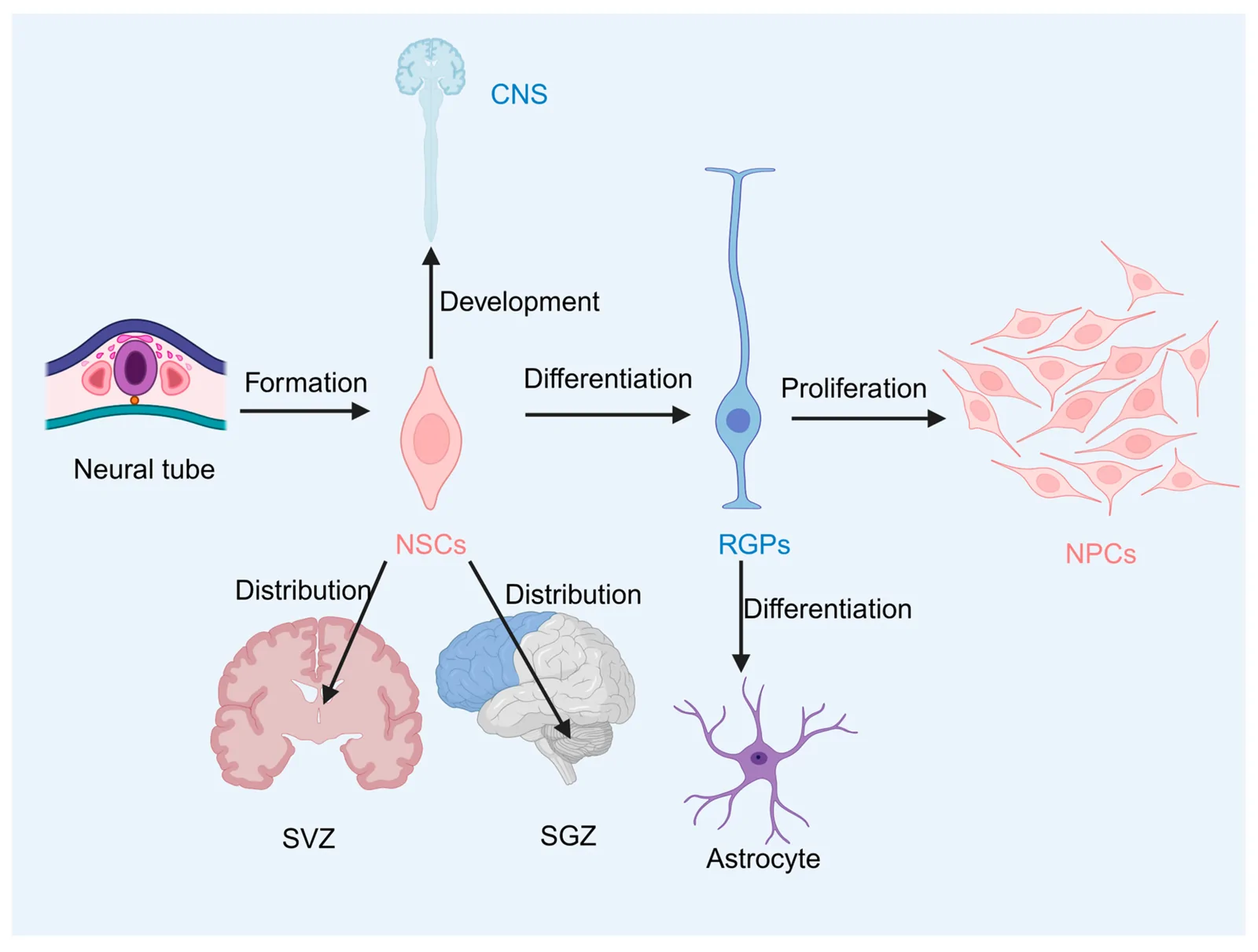
Neural stem cells (NSCs), also referred to as neuroepithelial cells, possess a remarkable ability to differentiate into radial glial progenitor cells (RGPs) and further proliferate into neural progenitor cell (NPC). These NPCs eventually mature into astrocyte-like cells or other specialized neural lineages. Notably, a subset of NSCs located along the neural tube plays a pivotal role in generating the central nervous system (CNS) during early development. In adults, NSCs persist in specific niches, including the subventricular zone (SVZ) of the lateral ventricles and the subgranular zone (SGZ) of the dentate gyrus within the hippocampus, where they contribute to ongoing neurogenesis and tissue homeostasis.

NSCs, also known as neuroepithelial cells (NECs), can differentiate into radial glial progenitor cells (RGPs) and proliferate into a pool of neural progenitor cells (NPCs) [29,30]. During the early stages of embryonic development, the nervous system develops from a neural plate made up of NECs, which expand the cell pool through symmetric division. The earliest form of embryonic NSCs originates from the division of NECs [31,32]. The CNS is formed during development by a small number of NSCs that line the neural tube [33]. At the same time, NSCs differentiate from neuroepithelial cells to RGPs, eventually remaining as astrocyte-like cells throughout the postnatal and adult brain [34,35,36]. In adult mammals, NSCs exist in the SVZ and SGZ (subgranular zone of the dentate gyrus in the hippocampus) [2]. RGPs present in the SGZ of the dentate gyrus can serve as both NPCs for neuronal generation and scaffolds for promoting neuronal migration during the development of the CNS. NSCs can also serve as a lifelong source for most neurons in the CNS (Figure 1) [37,38].

**Figure 2:**
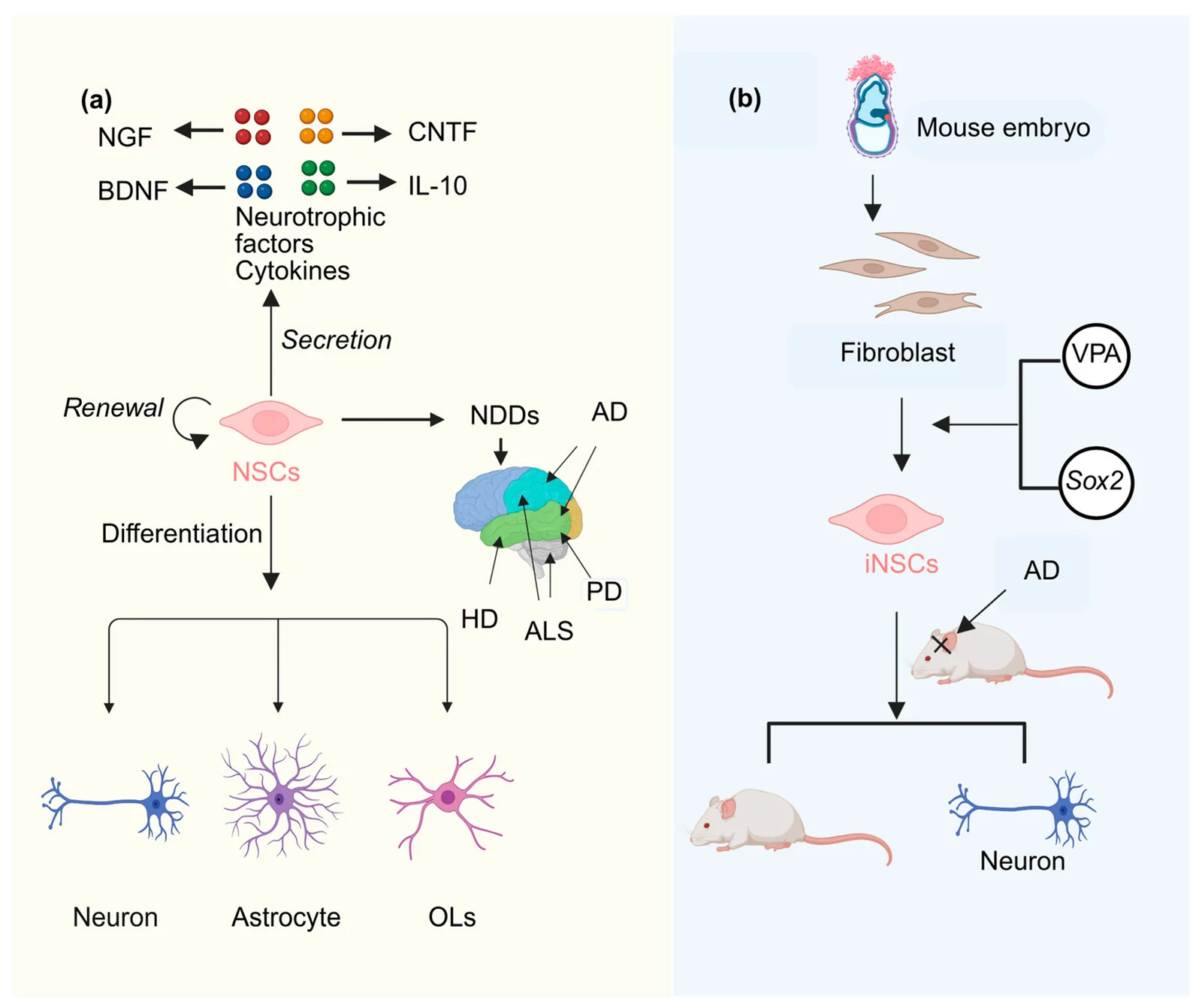
(**a**) NSCs can differentiate into neurons, astrocytes, and oligodendrocytes (OLs). Additionally, they are capable of releasing cytokines and neurotrophic factors, including Nerve Growth Factor (NGF) and Brain-Derived Neurotrophic Factor (BDNF), Ciliary Neurotrophic Factor (CNTF), and interleukin-10 (IL-10). Neural stem cells play a pivotal role in the treatment of neurodegenerative diseases (NDDs). NDDs encompass Alzheimer’s disease (AD), Parkinson’s disease (PD), Huntington’s disease (HD), and amyotrophic lateral sclerosis (ALS). (**b**) Valproic acid (VPA) combined with Sox2 can enhance the conversion efficiency of mouse embryonic fibroblasts into induced neural stem cells (iNSCs). Furthermore, after transplantation into the hippocampus of Amyloid Precursor Protein/Presenilin 1 (APP/PS1) AD mice, iNSCs can survive for an extended period, differentiate into functional neurons, and significantly improve cognitive deficits.

NSCs, as seed cells for treating nervous system diseases, possess the potential for self-renewal and multilineage differentiation. They are capable of differentiating into neurons, astrocytes and OLs [39]. NSCs can also release cytokines and neurotrophic factors to facilitate functional recovery after injury [40]. Specifically, they produce neurotrophic factors such as Nerve Growth Factor (NGF) and Brain-Derived Neurotrophic Factor (BDNF) [41], as well as cytokines including Ciliary Neurotrophic Factor (CNTF) [42] and interleukin-10 (IL-10) [43,44]. These factors collectively form the basis for NSCs-based therapies in central nervous system diseases [45]. In neuroscience research, the transplantation of NSCs for the treatment of neurological diseases has attracted widespread attention in recent years [46,47,48]. NSCs transplantation has been studied as a potential therapeutic approach, particularly for neurodegenerative diseases [49]. NDDs include Alzheimer’s disease (AD), Parkinson’s disease (PD), Huntington’s disease (HD), and Amyotrophic lateral sclerosis (ALS), among others [50]. It has been demonstrated that NSC transplantation can significantly improve cognitive function in transgenic models of AD (Figure 2a) [51,52]. However, directly obtaining neural stem cells from human donors is not practical. Instead, mouse fibroblasts can be directly reprogrammed into induced neural stem cells (iNSCs) [53]. Building on this finding, subsequent studies have shown that the combination of valproic acid (VPA) and the transcription factor Sox2 can enhance the conversion efficiency of mouse embryonic fibroblasts into induced neural stem cells (iNSCs). Furthermore, iNSCs transplanted into the hippocampus of APP/PS1 Alzheimer’s disease (AD) mice can survive for an extended period, differentiate into functional neurons, and significantly improve cognitive impairment (Figure 2b) [54].

## 3. The Effect of Microgravity on the Development and Regeneration of Neural Stem Cells

### 3.1. Effects of Microgravity on the Developmental Ability of Neural Stem Cells

**Figure 3:**
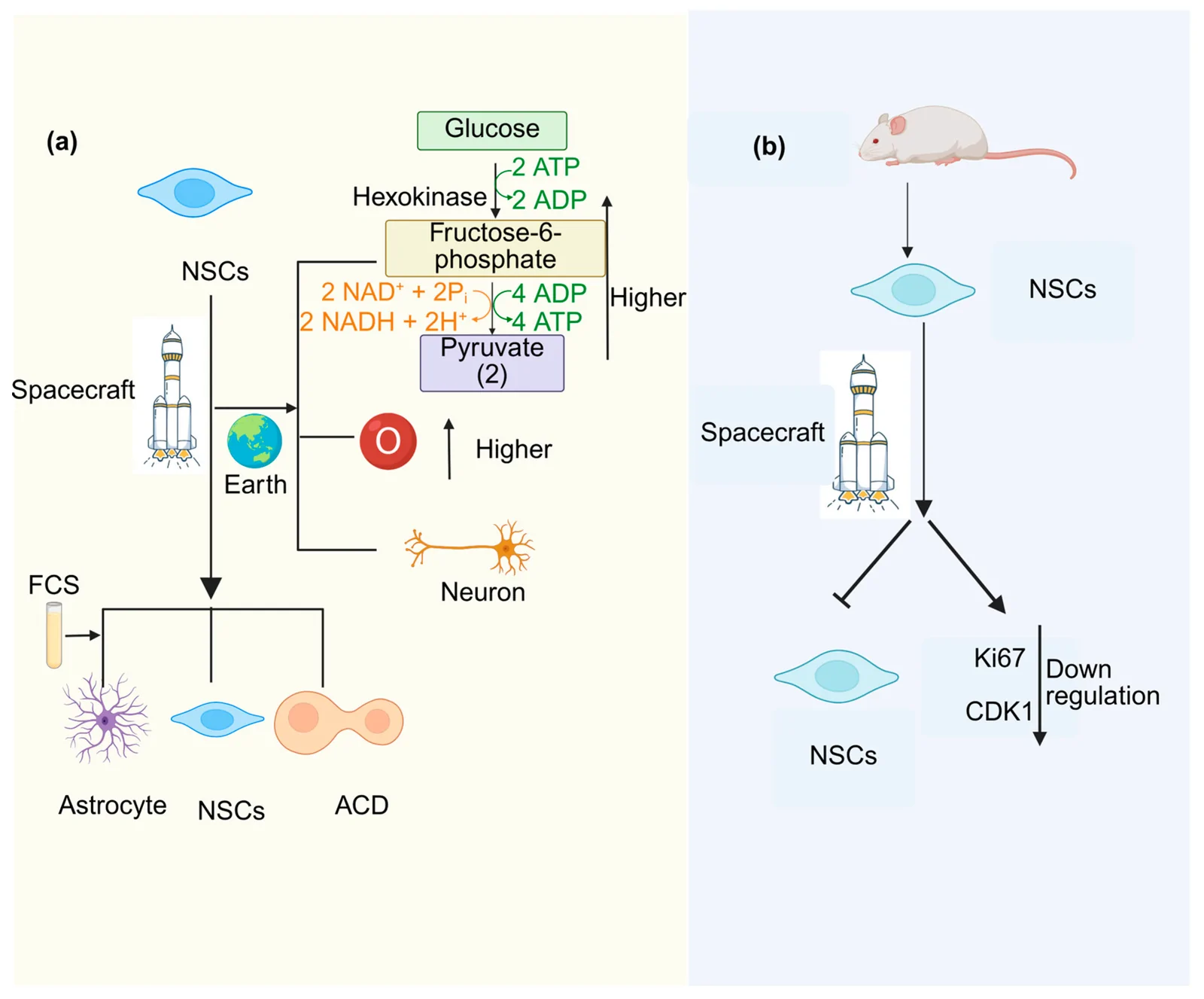
(**a**) NSCs not only proliferate successfully in space but also exhibit higher oxygen consumption and glycolytic capacity upon return to Earth compared to ground-based controls, while maintaining the ability to differentiate into young neurons. Under microgravity conditions, the number of NSCs increases, and astrocytes can also be induced with the supplementation of fetal calf serum (FCS). However, abnormal cell division (ACD) also occurs under space conditions. (**b**) The volume of neural spheres derived from rat NSCs decreases in space. The growth rate of cells slows down, and the expression of the cell proliferation marker Ki67, as well as Cyclin-dependent kinase 1 (CDK1), is downregulated during spaceflight.

Microgravity exerts divergent effects on NSCs proliferation, with outcomes varying markedly across experimental conditions. For instance, human NSCs cultivated for 39.3 days aboard the International Space Station maintained sustained proliferative capacity. Metabolic analysis showed that the oxygen consumption rate (OCR) and extracellular acidification rate (ECAR, glycolysis index) of NSCs in space environment were significantly higher than those in the ground control group. Additionally, human NSCs retain the ability to differentiate into young neurons under appropriate conditions [55]. Human NSCs cultured in space exhibit a proliferation rate seven times higher than ground control cells [56]. Observation of NSCs exposed to space reveals that although these cells have a higher proliferation rate than ground control cells, spaceflight exposes NSCs to abnormal cell division (ACD), particularly incomplete cytokinesis (ICD). Upon return to Earth, ICD occurred at 0.62% one week post-flight, whereas total ACD frequency (0.56%) remained comparable to ground controls (0.58%). By two weeks post-flight, however, both ICD (1.22%) and total ACD (1.68%) had increased significantly above ground controls—indicating a delayed manifestation of spaceflight-induced division abnormalities (Table 1) [25] At the same time, in culture medium supplemented with fetal bovine serum (FCS), human NSCs flown to space successfully induce the astrocyte phenotype (Figure 3a) [57].

However, NSC proliferation becomes dysregulated in microgravity environments, with studies reporting seemingly contradictory outcomes. Consistent with this cell cycle dysfunction, automated imaging during spaceflight revealed decreased neurosphere volume. Although these cells retained stem cell properties in space, their growth rate was reduced, accompanied by downregulation of the proliferation markers such as Ki67 and cyclin-dependent kinase 1 (CDK1). (Figure 3b) [26]. Silvano’s team simulated that microgravity can cause the cell cycle of mouse NSCs to stagnate in the S phase, inhibit cell proliferation, and maintain the simulated microgravity environment for 3 to 5 days [58]. This is different from the proliferation enhancement observed in human neural stem cells during long-term (39.3 days) spaceflight [55,56]. These contradictions may reflect differences in microgravity methods, species, and exposure time.

Microgravity can be achieved through simulated microgravity and real microgravity. However, real microgravity presents challenges such as high cost, long cycle, and poor reproducibility, making it difficult to implement on a large scale. As a result, researchers are increasingly focusing on simulated microgravity as an alternative approach. Currently, research on cultivating neural stem cells under simulated microgravity conditions primarily employs two methods: the Rotary Cell Culture System (RCCS) and the Rotating Wall Vessel (RWV).

Microgravity exerts context-dependent effects on NSC differentiation, with outcomes varying based on the culture platform and environmental cues. As multipotent precursors, NSCs retain the capacity for self-renewal and can generate both glial and neuronal lineages under appropriate conditions [59]. Under specific conditions, microgravity biases NSCs toward neuronal commitment in both 2D and 3D systems [60]. For instance, using an RWV bioreactor to simulate microgravity, researchers observed enhanced neuronal and astrocytic differentiation in rat embryonic NSCs/NPs following collagen embedding [61]. Conversely, NSCs cultured in the RCCS bioreactor derived from rat brain hemispheres exhibited inhibited differentiation of astrocytes when exposed to culture medium containing neurotrophic factor-3 (NT-3) [62].

Under actual spaceflight conditions, however, a distinct pattern emerged. Immunofluorescence analysis of post-flight samples revealed elevated levels of the mature neuronal marker microtubule-associated protein 2 (MAP2), reduced expression of the astrocytic marker glial fibrillary acidic protein (GFAP), and unchanged levels of the early neuronal marker *Tuj1* expression. Spaceflight biases NSCs toward neuronal rather than astrocytic fate commitment, with the specific outcome moderated by culture platform and differentiation cues [26]. Therefore, it can be inferred that the differences in NSC differentiation may be attributed to the method of microgravity simulation, which determines the source and location of neural stem cells.

**Table 1:** Contrasting effects of microgravity on neural stem cell proliferation across different experimental conditions.

Gravity Condition	Training Duration	Mode	Key Findings	References
Real space flight	39.3 days	Induced pluripotent human neural stem cells (CS83iCTR-33nxx)	proliferate	[55]
Real spaceflight International Space Station (ISS)		Induced pluripotent human neural stem cells (CS83iCTR-33n21)	Proliferation (seven times higher than ground proliferation)	[56]
Real spaceflight	1–2 weeks after returning	Induced pluripotent human neural stem cells (CS83iCTR-33n21)	abnormal cell division (ACD)	[25]
Real spaceflight (3D)		Rat NSC (rat telencephalon tissues)	decrease in neurosphere volume	[26]
RWV simulation (rotating wall bioreactor)	Up to 9 weeks	Rat embryonic NSC/NPs (embryonic day 13 (E13) dissected rat cortical neuroepithelium)	enhanced in their ability to generate neurons and astrocytes	[61]
RCCS simulation		Rat astrocyte	Differentiation inhibited (including NT-3 medium)	[62]

### 3.2. Effects of Microgravity on the Regeneration Ability of Neural Stem Cells

NSCs are endogenous precursors in the CNS with the capacity for self-renewal and multipotent differentiation into neurons and glial cells [63]. These properties have positioned NSC transplantation as a potential therapeutic strategy for NDDs [46,47,48]. Nevertheless, clinical application is hindered by three major challenges: poor graft survival, limited migratory capacity, and incomplete functional integration at injury sites. While endogenous NSCs can migrate toward CNS lesions following injury, this endogenous repair response is typically insufficient in both scope and efficiency [64].

NSCs are multipotent stem cells capable of differentiating into neuronal and glial lineages, and they have shown potential to contribute to spinal cord repair in preclinical models [65]. In a rat spinal cord injury model, NSCs cultured under simulated microgravity (RCCS) exhibited enhanced migratory capacity two weeks post-transplantation. Cells migrated away from the implantation site, while static-cultured cells remained confined. NT-3 treatment further potentiated this effect, resulting in more extensive migration and greater distances covered compared to RCCS alone. These findings indicate that microgravity preconditioning synergizes with neurotrophic factors to improve NSC migration in vivo [62].

When NSCs were cultured on a collagen sponge for 7 days, a significant increase in the percentage of NeuN- and *Sox2*-positive cells was observed in the RCCS group. In contrast, the percentage of *Ki67*-positive cells slightly decreased. These findings suggest that a microgravity environment promotes the self-renewal capacity and neurogenic potential of 3D cultured NSCs. Furthermore, rats implanted with NSC-laden collagen sponge scaffolds exhibited better exercise recovery compared to those implanted with only collagen sponge scaffolds or the control group. At 8 weeks post-surgery, the average Basso–Beattie–Bresnahan (BBB) score of rats in the RCCS group reached 8.3 ± 0.6, which was significantly higher than that of the control group (2.0 ± 1.0), the collagen sponge group (2.8 ± 1.0), and the static culture group [66].

## 4. The Regulatory Mechanism of Microgravity on the Development and Regeneration of Neural Stem Cells

### 4.1. The Regulatory Mechanism of Microgravity on the Development of Neural Stem Cells

#### Differential Regulation of Core Signaling Pathways

As space exploration advances, astronauts inevitably experience microgravity conditions [67]. Emerging evidence suggests that microgravity regulates NSC fate by reshaping a multi-path signaling network. Specifically, the Hippo and BMP pathways collaborate to establish the developmental trajectory of NSC, while Notch-mediated inhibition and Wnt-mediated activation provide additional layers of fate control.

Transcriptome analysis of 3D cultured rat neural stem cells from the SJ-10 satellite space mission (12 days) and ground control revealed significant changes induced by spaceflight. In proliferating cells, 1589 differentially expressed genes (DEGs) were identified, alongside 3279 DEGs in differentiated cells. Compared with the ground group, the spaceflight group exhibited decreased levels of 93 microRNAs (miRNAs) during proliferation and increased levels of 90 miRNAs. However, the absence of pathway-specific intervention studies precludes determining which molecular changes are necessary or sufficient for the observed phenotypes [26]. In proliferating NSCs, spaceflight upregulated transcripts for established stemness regulators, including *Rest1* and Krüppel-like factor 4 (*Klf4*), concomitant with increased *Pax6*, *Sox2* (qPCR-validated), and *Notch1* expression [26]. Conversely, under differentiation conditions, the same spaceflight exposure accelerated neuronal maturation, evidenced by elevated mature neuronal marker MAP2 and reduced astrocytic marker GFAP [26]. Immunofluorescence confirmed elevated MAP2 protein levels (*p* < 0.01) but unchanged early neuronal marker *Tuj1*—indicating that spaceflight accelerates maturation of already-committed neurons rather than driving de novo neuronal commitment.

**Figure 4:**
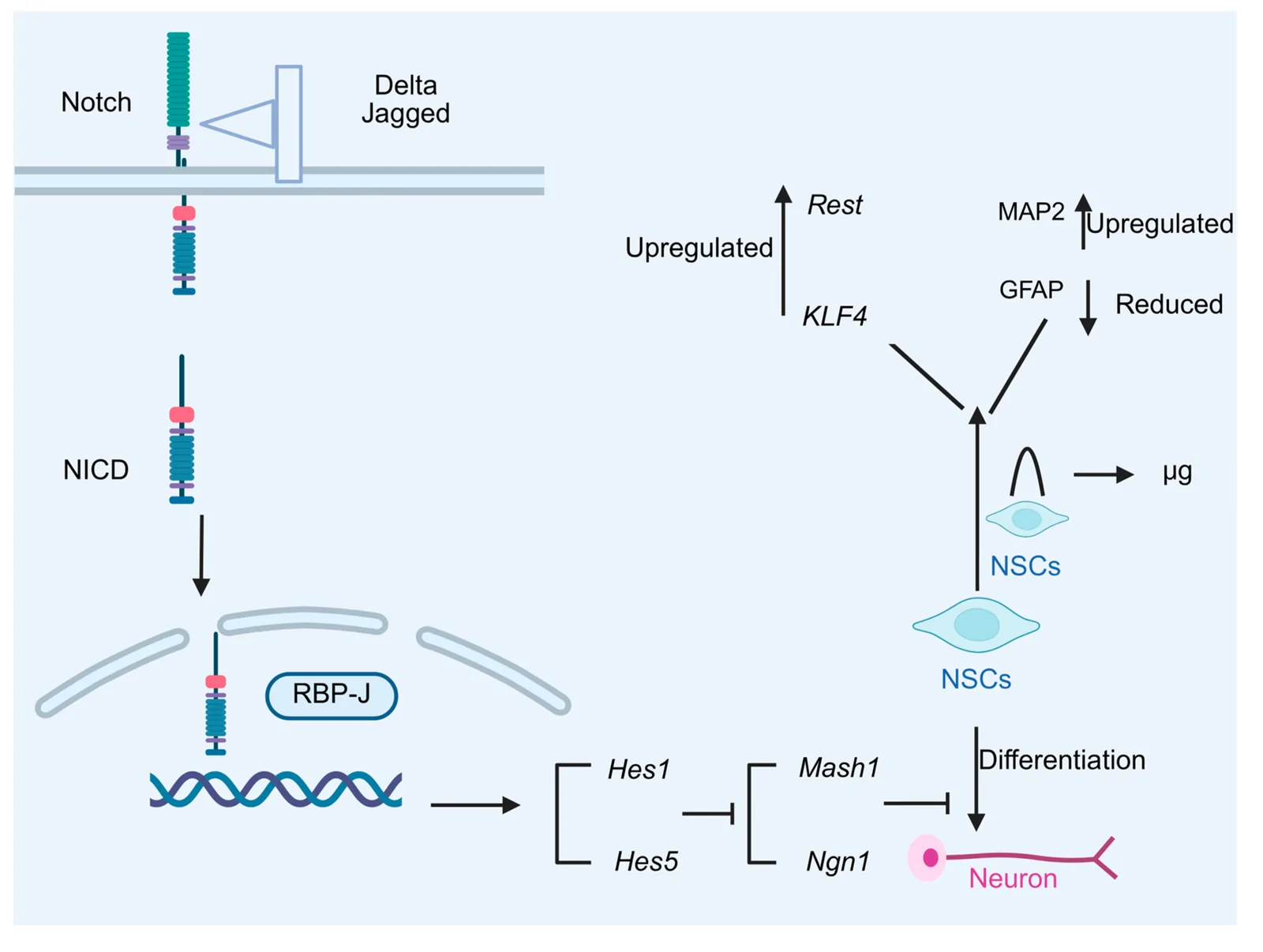
Under microgravity conditions, the expression of stemness-related genes (such as Repressor Element 1-Silencing Transcription Factor (*Rest*) and Krüppel-like factor 4 (*Klf4*)) in neural stem cells is upregulated in the space group. Simultaneously, neural stem cells exhibit a tendency to differentiate into neurons under microgravity, evidenced by an increase in the expression of the mature neuron marker microtubule-associated protein 2 (MAP2), and a concomitant decrease in the expression of astrocyte marker glial fibrillary acidic protein (GFAP). Under normal gravity conditions, Notch receptors bind to their ligands and are activated, leading to the dissociation of the Notch intracellular domain (NICD) from the cell membrane. NICD subsequently translocates into the nucleus, where it binds to the transcription factor Recombination Signal Binding Protein J (RBP-J), forming a functional complex. This complex activates the transcription and expression of downstream repressive genes, such as Hairy and Enhancer-of-split homologues 1 and 5 (*Hes1* and *Hes5*). The expression of these repressive genes inhibits the activity of neurogenic transcription factors, including Mammalian Achaete-Scute Homolog 1 (*Mash1*) and neurogenin1 (*Ngn1*), thereby blocking the differentiation process of NSCs into neurons.

The Notch signaling pathway regulates NSCs differentiation along the neuronal to glial “switch” route and is a critical determinant of NSC fate during development [68,69]. Under normal circumstances, Notch receptors bind to their ligands (Delta/Jagged), undergo γ-secretase-dependent cleavage to release the Notch Intracellular Domain (NICD), which translocates to the nucleus and converts RBP-J from a transcriptional repressor to an activator by recruiting MAML coactivators. This tripartite complex drives Hairy and Enhancer of *Split 1* and 5 (*Hes1*/*Hes5*) expression, which in turn inhibits proneural genes to prevent neuronal differentiation [70,71,72]. The expression of these inhibitory genes suppresses the activity of neurogenic transcription factors such as Mammalian Achaete-Scute Homolog 1 (*Mash1*) and neurogenin1 (*Ngn1*), preventing the development of NSCs into neurons [73] (Figure 4). Under simulated microgravity conditions, the Notch signaling pathway exhibits altered activity that may influence NSC differentiation kinetics [74]. Whether this promotes neuronal or glial lineage commitment remains unresolved due to conflicting reports and the correlative nature of existing evidence.

**Figure 5:**
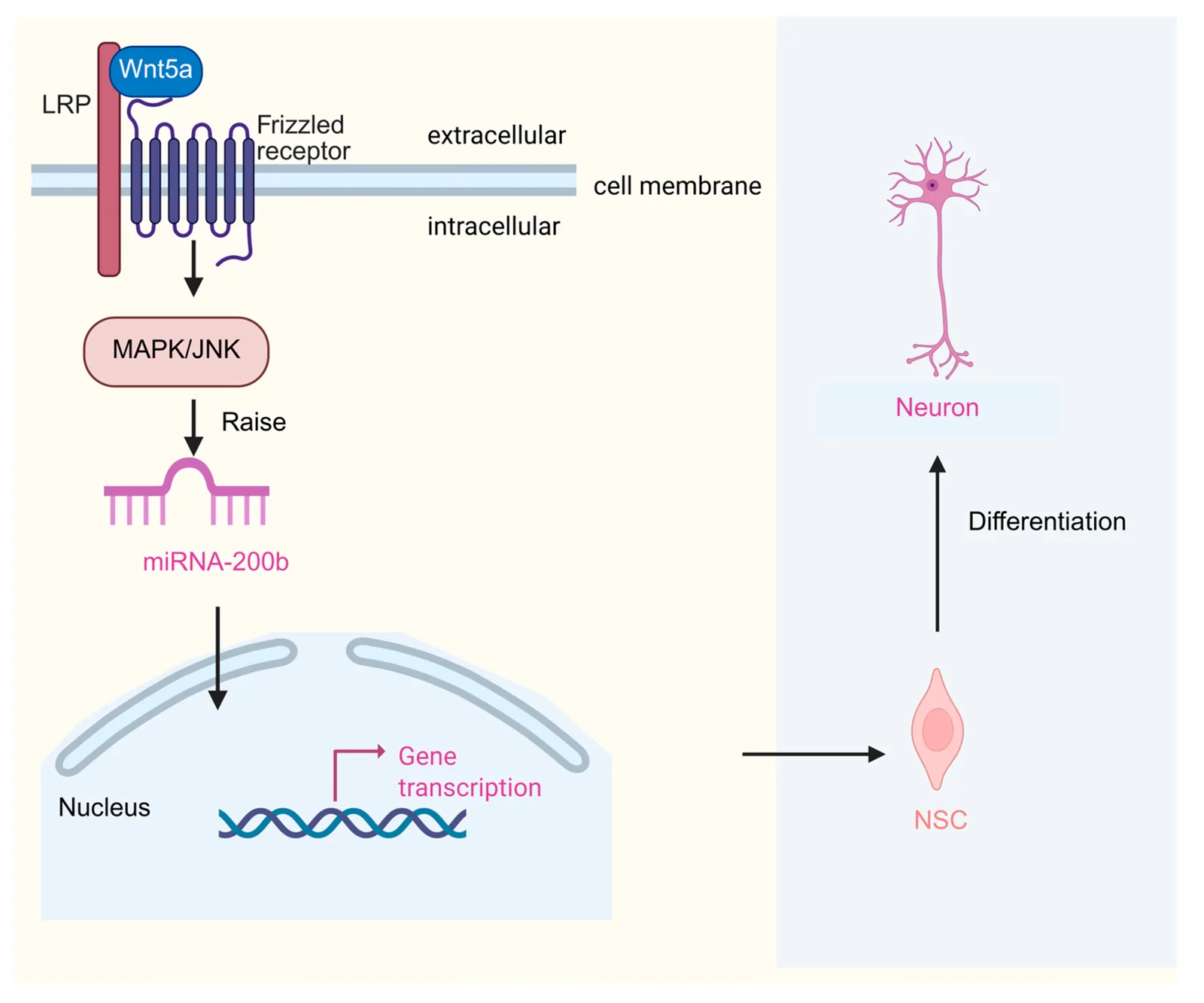
Wnt5a enhances the expression of miRNA-200b via the mitogen-activated protein kinase/c-Jun N-terminal kinase (MAPK/JNK) signaling pathway, thereby facilitating the differentiation of NSCs into neurons.

The dynamic adjustment of the Wnt signaling pathway further refines differentiation regulation, with each specific mechanistic claim supported by corresponding experimental evidence, along with unresolved research gaps that warrant further investigation. In the dentate gyrus of adult mice, a decrease in Wnt7a expression accelerates NSCs withdrawal from the cell cycle, leading to a significant reduction in NSCs proliferation. The number of BrdU-positive cells in neural stem cells with *Wnt7a* deficiency is significantly reduced [75]. *Wnt3* promotes the fate commitment of adult SGZ neurons and the proliferation of neural precursors [76,77]. *Wnt5a* is a key regulatory factor in the non-classical Wnt signaling pathway. *Wnt5a* upregulates the expression of miRNA-200b through mitogen-activated protein kinase/c-Jun N-terminal kinase (MAPK/JNK) signaling, increasing NSCs differentiation into neurons (Figure 5) [78]. Some microRNAs (miRNAs) indirectly activate the Wnt signaling pathway by targeting negative regulatory factors of the Wnt signaling pathway, thereby promoting the proliferation and differentiation of NSCs [26]. In experiments simulating microgravity, certain signal cascades of the β-catenin signaling pathway in the gut of *Caenorhabditis elegans* still exist and play a role, potentially playing a vital part in the gut’s response [79].

Beyond Notch and Wnt pathways, microgravity may modulate auxiliary signaling pathways, including Hippo. Temporary YAP1 activation can aid cell survival, proliferation, and regeneration [80]. Simulating a microgravity environment can interfere with the number and function of NSCs and progenitor cell subpopulations, altering Hippo and BMP signaling in brain organoids [81]. Microgravity simulation using RCCS induces β-adrenergic receptors, upregulates cyclic adenosine monophosphate (cAMP) formation, and activates protein kinase A (PKA) and cAMP response element-binding protein (CREB). Mitochondrial mass and Adenosine triphosphate (ATP) levels increase due to microgravity, indicating that microgravity can also promote the proliferation of NSCs by improving mitochondrial function [82].

The findings underscore the complexity of microgravity’s influence on NSC behavior, highlighting the interplay between multiple signaling pathways. While the Hippo and BMP pathways appear to guide NSC development, the opposing roles of Notch and Wnt signaling add nuanced layers of regulation. This dual-layered control mechanism may explain why spaceflight induces both enhanced stemness in proliferating NSCs and accelerated maturation in differentiating neurons.

### 4.2. Regulation Mechanism of Microgravity on Neural Stem Cell Regeneration

#### Improvement of the Extracellular Matrix and Survival Environment

Extracellular matrix (ECM) remodeling is becoming a key regulator of neural maturation and axonal extension. The ECM is critical for sustaining NSCs’ stemness. Under simulated microgravity conditions, it can regulate the interaction between human neural stem/precursor cells (hNSPCs) and the extracellular matrix based on laminin, leading to cell shedding and transcriptional regulation of genes involved in cell adhesion and stress responses. Placing human neural stem precursor cells (hNSPC) in a random positioning machine (RPM) device for 24 h showed that reducing gravity counteracts the interaction between cells and the extracellular matrix, inducing morphological changes and feedback regulation of genes involved in local adhesion and the cell cycle. After 6 h of simulated microgravity, transcription factor Kruppel-like factor 6 (KFL6) was upregulated along with Krüppel-like factor 13 (KFL13). Global transcriptome analyses under simulated microgravity for 24 h showed that RNA-Binding Motif protein 3 (RBM3), Myocardial Infarction-Associated Transcript (MIAT), and Growth Arrest and DNA Damage-inducible 45 Gamma (GADD45G) were repeatedly upregulated [83].

In a microgravity environment, neural stem cells exhibit unique growth characteristics and tend to aggregate together to form 3D structures. This structure not only increases mechanical, structural, and chemical interactions between cells and the ECM but also creates a milieu more similar to the physiological environment in vivo for NSCs’ growth [13]. Extracellular vesicles derived from NSCs are rich in specific miRNAs, which mediate multiple functions under physiological and pathological conditions [84]. Furthermore, the study revealed that exosomes derived from neural progenitor cells are enriched in miR-21a, a miRNA that can stimulate the differentiation of neural stem cells into neurons in vitro. This provides evidence that extracellular vesicles derived from NSCs promote the development of NSCs through miRNAs [85]. Specifically, extracellular vesicles secreted by NSCs can carry miRNAs (such as miR-374-5p), which can target neurons at the site of injury, inhibit apoptosis by activating autophagy flux, and promote the recovery of nerve injury. miR-374-5p in extracellular vesicles activates the autophagy pathway and inhibits apoptosis by targeting serine/threonine-protein kinase 4 (*STK-4*) gene [86]. Instead of NSCs, glial cells are one of the byproducts of differentiation. Extracellular vesicles (including exosomes) released by damaged glial cells in simulated spatial environments can protect neurons to a certain extent, with exosomes playing a critical role in this process [87].

## 5. Future Research Directions and Summary

In this study, we summarized the effects of microgravity on the development and regeneration ability of NSCs. Our findings reveal that real spaceflight significantly enhances NSC proliferation while reducing neurosphere volume. In contrast, simulated microgravity using a rotating wall vessel (RWV) system enhances the ability of NSCs to generate neurons and astrocytes. However, in a rotary cell culture system (RCCS), differentiation is inhibited, even in the presence of differentiation-promoting medium. Notably, the degree of damage to NSCs cultured under simulated microgravity for 24, 48, and 72 h was higher than that observed under static conditions. Conversely, after 96 and 120 h of rotation, the degree of damage decreased, indicating that NSCs gradually adapt to microgravity [88]. This “promotion-inhibition” effect of microgravity on NSC proliferation represents a phenotype difference influenced by key factors such as experimental conditions, cell sources, and observation time, warranting further investigation.

Regarding differentiation, current evidence suggests that microgravity activates the Wnt/β-catenin signaling pathway, promoting the neuronal differentiation of NSCs. However, the mechanisms underlying cross-pathway interactions remain unclear and require additional research.

For clinical translation, these findings suggest two potential applications. First, the enhanced 3D spheroid formation and sustained viability of NSCs under microgravity could be leveraged to develop scalable bioreactor systems for producing high-quality NSCs for transplantation in PD and AD. Second, understanding the adaptive mechanisms NSCs employ after 96 h—such as stress response pathways—may reveal novel cytoprotective targets to enhance graft survival in hostile host environments. Future experiments should validate whether microgravity-expanded NSCs maintain therapeutic efficacy upon transplantation into neurodegenerative disease models. Additionally, long-term in vivo monitoring should assess their tumorigenic potential, and optimal culture durations balancing expansion with phenotypic stability should be determined.

Overall, microgravity affects NSCs through multi-level processes involving cell morphology, signal transduction, gene expression, and microenvironmental changes. As technology advances, further research should focus on the cellular mechanisms, differentiation characteristics, and therapeutic applications of NSCs in space medicine and neurodegenerative disease treatment, ultimately providing evidence-based strategies for clinical implementation.

## Data Availability

No new data were created or analyzed in this study.

## Abbreviations

The following abbreviations are used in this manuscript:

NSCs	Neural Stem Cells
iNSCs	Induced Neural Stem Cells
OLs	Oligodendrocyte
SGZ	Subgranular Zone
SVZ	Subventricular zone
NDDs	Neurodegenerative Disease
NPCs	Neural progenitor cell
RGPs	Radial glial progenitor
ACD	Abnormal cell division
BMP	Bone Morphogenetic Protein
CDK1	Cyclin-dependent kinase 1
ECM	Extracellular matrix
FCS	Fetal Calf Serum
GFAP	Glial fibrillary acidic protein
MAP2	Microtubule-associated protein 2
RWV	Rotating Wall Vessel
VPA	Valproic acid
AD	Alzheimer’s Disease
ALS	Amyotrophic Lateral Sclerosis
APP/PS1	Amyloid Precursor Protein/Presenilin 1
ATP	Adenosine Triphosphate
BBB	Basso-Beattie Bresnahan
cAMP	cyclic Adenosine Monophosphate
CNS	Central Nervous System
CREB	cAMP Response Element-Binding protein
DEGs	Differentially Expressed Genes
ECAR	Extracellular Acidification Rate
GADD45G	Growth Arrest and DNA Damage-inducible 45 Gamma
HD	Huntington’s Disease
Hes1/Hes5	Hairy and Enhancer of Split 1 and 5
hNSPCs	human Neural Stem/Precursor Cells
ICD	Incomplete Cell Division (or Incomplete Cytokinesis)
ISS	International Space Station
Klf4	Krüppel-like factor 4
KFL6	Krüppel-like factor 6
MAML	Mastermind-Like
Mash1	Mammalian Achaete-Scute Homolog 1
miRNAs	microRNAs
MIAT	Myocardial Infarction-Associated Transcript
Ngn1	Neurogenin 1
NeuN	Neuronal Nuclei
NICD	Notch Intracellular Domain
NT-3	Neurotrophin-3
OCR	Oxygen Consumption Rate
Pax6	Paired box 6
PD	Parkinson’s Disease
PKA	Protein Kinase A
qPCR	Quantitative Polymerase Chain Reaction
RBP-J	Recombination Signal Binding Protein-Jκ
Rest	RE1-Silencing Transcription Factor
RBM3	RNA-Binding Motif protein 3
RPM	Random Positioning Machine
Sox2	SRY-box transcription factor 2
STK-4	Serine/Threonine Kinase 4
Yap1	Yes-associated protein 1
CNTF	Ciliary Neurotrophic Factor
2D	two-dimensional
3D	three-dimensional
NGF	Nerve Growth Factor
BDNF	Brain-Derived Neurotrophic Factor
IL-10	Interleukin-10
KLF13	Krüppel-like factor 13
